# Cepharanthine loaded nanoparticles coated with macrophage membranes for lung inflammation therapy

**DOI:** 10.1080/10717544.2021.2009936

**Published:** 2021-12-06

**Authors:** Caihong Lu, Jinpeng Zheng, Yaning Ding, Yuanyuan Meng, Fangyun Tan, Wei Gong, Xiaoyang Chu, Xiaolong Kong, Chunsheng Gao

**Affiliations:** a School of Pharmacy, Guangxi Medical University, Nanning, P. R. China; b State Key Laboratory of Toxicology and Medical Countermeasures, Beijing Institute of Pharmacology and Toxicology, Beijing, P. R. China; c School of Pharmaceutical Engineering, Shenyang Pharmaceutical University, Shenyang, P. R. China; d Department of Stomatology, The Fifth Medical Center of PLA General Hospital, Beijing, P. R. China

**Keywords:** Macrophage membrane, bionic nanocarrier, cepharanthine, acute lung injury, inflammation

## Abstract

Acute lung injury (ALI) is a disease associated with suffering and high lethality, but to date without any effective pharmacological management in the clinic. In the pathological mechanisms of ALI, a strong inflammatory response plays an important role. Herein, based on macrophage ‘homing’ into inflammation sites and cell membrane coating nanotechnology, we developed a biomimetic anti-inflammation nanosystem (MM-CEP/NLCs) for the treatment of ALI. MM-CEP/NLCs were made with nanostructured lipid carriers (NLCs) coated with natural macrophage membranes (MMs) to achieve effective accumulation of cepharanthine (CEP) in lung inflammation to achieve the effect of treating ALI. With the advantage of suitable physicochemical properties of NLCs and unique biological functions of the macrophage membrane, MM-CEP/NLCs were stabilized and enabled sustained drug release, providing improved biocompatibility and long-term circulation. *In vivo*, the macrophage membranes enabled NLCs to be targeted and accumulated in the inflammation sites. Further, MM-CEP/NLCs significantly attenuated the severity of ALI, including lung water content, histopathology, bronchioalveolar lavage cellularity, protein concentration, and inflammation cytokines. Our results provide a bionic strategy via the biological properties of macrophages, which may have greater value and application prospects in the treatment of inflammation.

## 1. Introduction

Acute lung injury (ALI) is a diffuse inflammatory injury to lung epithelium/endothelium caused by various factors, such as septicemia (Kumar, 2020), bacterial infections (Lucas et al., 2020), and viral infections (Zhang et al., 2020). Severe ALI can develop into acute respiratory distress syndrome (ARDS), which has the characteristics of high morbidity and high mortality (Inoue et al., 2007; Matthay et al., 2012). Severe Acute Respiratory Syndrome Coronavirus 2 (SARS-CoV-2) causes coronavirus disease-19 (COVID-19) which is a newly-recognized infectious disease. It has rapidly transmitted and become a major concern all over the world. Based on clinical features, pathology, the pathogenesis of acute respiratory disorder induced by either highly homogenous coronaviruses or other pathogens, the evidence suggests that excessive inflammation, oxidation, and an exaggerated immune response very likely contribute to COVID-19 pathology (Zhang et al., 2020). This leads to a cytokine storm and subsequent progression to ALI/ARDS and often death.

Cepharanthine (CEP) is a biscoclaurine alkaloid extracted from *Stephania cepharantha* Hayata (Murakami et al., 2000; Kim et al., 2019). CEP has been shown to have a variety of effects, such as raising leukocyte (Furusawa & Wu, 2007), anti-inflammatory (Kao et al., 2015), anti-cancer (Yu et al., 2016), and enhancing immune regulation (Uto et al., 2011). Recent data in the literature showed that CEP can suppress nuclear factor-kappa B (NF-κB) activation, lipid peroxidation, nitric oxide production, cytokine production, and expression of cyclooxygenase, all of which are crucial to viral replication and inflammatory response (Rogosnitzky et al., 2020). Against SARS-CoV-2 and homologous viruses, CEP predominantly inhibits viral entry and replication *in vitro* (Ayele et al., 2021). Therefore, CEP is a promising candidate for the treatment of COVID-19 (Fan et al., 2020; McKee et al., 2020). However, a large dose (6–12 tablets per day) is required for current commercially available tablets. And CEP has poor solubility and is not easily absorbed by oral administration, resulting in low bioavailability (Deng et al., 2017). In addition, After the administration of the ordinary injection, CEP is quickly eliminated by the body, which has the problem of a short half-life. To effectively achieve a slow-release, reduce the side effects of the drugs, improve the bioavailability of the drugs, and transport anti-infect medicine selectively to inflammation tissues, lung targeted delivery is a promising therapeutic strategy.

Nanoparticles coated with biologically derived cell membranes have recently attracted attention for their cell-like simulation potential and natural targeting capabilities *in vivo* (Zhang et al., 2018; Gao et al., 2020; Rao et al., 2020). Macrophages are a type of white blood cells that find and engulf cellular debris, cancer cells, and foreign substances, and have the natural ability to be recruited to inflammation areas of the body (Rao et al., 2017; Han et al., 2020). In previous studies, macrophage membrane-coated nanoparticles have been used in the treatment of various diseases related to inflammation, such as cancer (Cao et al., 2016; Meng et al., 2018; Zhang et al., 2018), rheumatoid arthritis (Li et al., 2019), atherosclerosis (Wang et al., 2021) and aortic dissection (Liu et al., 2021). The innate inflammation-directed chemotactic ability of macrophages could drive the vector to accumulate in inflammatory tissue, showing superior targeting ability (Hu et al., 2020). In addition, the macrophage membrane-coated nanoparticles can also block the exposure of exogenous materials and avoid the uptake of the reticuloendothelial system (RES) to achieve the purpose of long-term circulation in the body (Liu et al., 2020). Therefore, a macrophage membrane-coated drug delivery system may be a potential new strategy for the treatment of inflammation and other related diseases.

Here, we explored the use of macrophage membranes to wrap the nanoparticles to enhance the accumulation of nanoparticles in the lungs, so as to attenuate acute lung injury. Specifically, first, a biomimetic drug delivery system was constructed by wrapping macrophage membranes isolated from a monocyte/macrophage cell line of RAW264.7 cells with nanostructured lipid carriers loading with CEP. Then, the biomimetic nanoparticles were injected into ALI mice by tail vein injection and recruited to the lung inflammation sites. Finally, the anti-inflammatory ability of the biomimetic nanoparticles was evaluated in the acute lung injury model.

## 2. Material and methods

### 2.1. Materials

Cepharanthine was purchased from Saen Chemical Technology Co., Ltd. (Shanghai, China). Labrafac lipophile WL 1349 was supplied by Gattefosse (Lyon, France). Soy Lecithin and coumarin-6(Cou6) were purchased from Yuanye Bio-Technology Co., Ltd. (Shanghai, China). Poloxamer188 was supplied by BASF (Ludwigshafen, Germany). 1,1′-Dioctadecyl-3,3,3′,3′-tetramethylindotricarbocyanine iodide (DiR) was purchased from Dalian Meilun Biotechnology Co., Ltd. (Dalian, China). Hoechst 33258 and all kinds of enzyme-linked immunosorbent assay (ELISA) kits were purchased from Solarbio (Beijing, China). 1,1′-Dioctadecyl-3,3,3′,3′-tetramethylindocarbocyanine perchlorate (DiI) and 4′,6-diamidino-2-phenylindole (DAPI) were purchased from Beyotime Institute of Biotechnology Co., Ltd. (Jiangsu, China). 3-(4,5-dimethyl-2-thiazolyl)-2,5-diphenyl-2-H-tetrazolium bromide (MTT) was purchased from Sigma-Aldrich (Louis, USA).

### 2.2. Cell culture and animal experimentation

RAW264.7 cells and human umbilical vein endothelial (HUVEC) cells purchased from the iCell Bioscience Inc. (Shanghai, China) were maintained in a culture medium consisting of Dulbecco’s modified Eagle’s medium (DMEM) supplemented with 10% fetal bovine serum (FBS) and 1% penicillin-streptomycin. The cells were maintained in a 37 °C humidified incubator in a 5% CO_2_ atmosphere.

Male Kunming (KM) mice (20–25 g) were provided by Vital River Laboratories (Beijing, China). All animal experiments complied with the code of ethics in research, training, and testing of drugs issued by the Animal Care and Use Ethics Committee in Beijing Institute of Pharmacology and Toxicology.

### 2.3. Preparation of nanoparticles

NLCs were prepared by solvent evaporation-ultrasonic dispersion method (Han et al., 2020). In brief, determined amount of soy lecithin and WL 1349 were melted in ethanol at 75 °C to form an organic phase. An amount of CEP or hydrophobic probe (Cou6 or Cy5.5) was added to the oil fraction. Aqueous phase consisting of Poloxamer 188 and sodium deoxycholate that had been dispersed in double-distilled water was also heated to 75 °C. The heated oil phase was injected into the Aqueous phase by drop by drop followed by magnetic stirring for approximately 1 h under 75 °C. The solution was then treated with cell ultrasound for 5 min (300 W, sonicated 2 s, stopped 5 s). The NLCs were obtained and stored at 4 °C. DiI or DiR labeled NLCs were obtained by adding free DiI or DiR to the nanosystem and stirring for 30 min in the dark. Free DiI and DiR were removed by dialysis for 12 h in the dark.

The CEP encapsulation efficiency (EE) of the formulation was determined by the ultrafiltration method using HPLC to quantify the CEP in NLCs (Li et al., 2017)

$$(\text{EE}\%=\;(W_{\text{total CEP}}-W_{\text{free CEP}})/W_{\text{total drug}}\times 100\%;\;\text{EE}\;=\;93.18\;\pm \;1.57\%).$$

### 2.4. Macrophage membrane derivation

The macrophage membranes were prepared as previously described with slight modification (Zhang et al., 2021). When RAW264.7 cells density reached 80–90%, the cells were harvested by centrifugation at 800 *g* for 5 min and washed three times with phosphate-buffered solution. Cells were then resuspended in ice-cold 0.25 × PBS containing 30 mM Tris-HCl, 0.5 mM EDTA, and a protease inhibitor cocktail. Cells were then disrupted by ultrasound for 2 min. Then, the obtained mixture was centrifuged (20,000 × *g*, 20 min, 4 °C, and 100,000 × *g*, 30 min, 4 °C) to collect the macrophage membranes. The protein content in the macrophage membranes was determined by the BCA protein assay.

### 2.5. Preparation of MM-CEP/NLCs

MM-CEP/NLCs were fabricated by coating CEP/NLCs with macrophage membranes by a direct extrusion method. Macrophage membranes were mixed with CEP/NLCs and ultrasonicated for 2 min (100 W; sonicated 2 s, stopped 5 s) in an ice bath. The mixture was then extruded 12 times through a 400 nm polycarbonate porous membrane using a mini extruder (Avanti Polar Lipids, AL, USA) to obtain MM-CEP/NLCs.

### 2.6. Characterization of the nanoparticles

The size, size distribution, and zeta potential of the prepared formulations were determined by dynamic light scattering (DLS) (Litesizer 500, Anton Parr, Austria). The morphology of nanoparticles was characterized via transmission electron microscopy (TEM) (HITACHI, H-7650, Japan). Prepared formulations in a solution containing 10% FBS were kept at 37 °C for the analysis of stability by measurement of particle diameter within 72 h.

### 2.7. In vitro release profile

The drug release from CEP/NLCs and MM-CEP/NLCs was studied separately using a dialysis method. Briefly, dialysis bags (MWCO: 8–14 kDa) with 2 mL of CEP-loaded formulations was immersed into 30 mL of phosphate-buffered saline (0.1 M, pH 7.4, containing 0.5% tween-80) at 37 °C. 2 mL of release medium was withdrawn at different time intervals and replaced with an equivalent volume of fresh PBS at 37 °C. The cumulative amount of CEP released was quantified by HPLC at 283 nm.

### 2.8. Protein determination

The protein profiles in the macrophage membranes and formulations were analyzed by Sodium dodecyl sulfate-polyacrylamide gel electrophoresis (SDS-PAGE) (Rao et al., 2017). The membrane protein was extracted from the macrophage membrane and MM-CEP/NLCs with RIPA lysis buffer (moderate, Beyotime, China) and further measured by an electrophoresis assay.

Furthermore, the CD44 and CD11b contents in macrophage membranes, NLCs, and MM-NLCs were determined by Western blot analysis (Gao et al., 2020). First, the total protein was extracted by Protein Extraction Kits (GenePool/GPP1815) and used for measurements. Samples underwent electrophoresis by 10% SDS-polyacrylamide gel and were transferred onto nitrocellulose membranes (Millipore, MA, USA). Then, the samples were treated with primary antibodies against CD44 (anti-CD44 antibody, ab157107, Abcam) and CD11b (anti-CD11b antibody, ab133357, Abcam), followed by the secondary antibody conjugated with horseradish peroxidase. Blots were visualized using ECL-Plus according to the manufacturer’s instructions.

### 2.9. Colocalization study

Fluorescent dye Cy5.5 was encapsulated into the core of NLCs, whose preparation procedure was similar to the process of the CEP loading experiment. The MMs were stained using DiI. Then, the DiI-labeled MMs were coated onto the Cy5.5 labeled NLCs by the same method of synthesis of MM-NLCs. For the colocalization study, HUVEC cells were maintained in DMEM supplemented with 10% FBS and cultured at 37 °C with 5% CO_2_. Then, dye-labeled MM-NLCs were added to the HUVEC cells. After incubation for an additional 2 h, the cells were washed with PBS three times, fixed with tissue fixative for 15 min, and then the nuclei of the cells were stained with Hoechst 33258. The cells were visualized by confocal microscopy.

### 2.10. Hemolysis assay of empty nanosystems

Erythrocytes (0.2 mL) from healthy rats were seeded into 96-well plates. Next, the cells were incubated with 0.9% (w/v) sodium chloride solution (Negative control), double distilled water (Positive control), empty NLCs, or empty MM-NLCs. After incubation at 37 °C for 1 h, the cells were centrifuged, and then the supernatants of the sample were measured at 540 nm using a microplate reader (Tecan, Spark, Austria) (Bardania et al., 2019). The equation for the rate of hemolysis is as follow:

$$\text{Hemolysis}\;(\%)=\frac{\left(OD_{\mathrm{sample}}-OD_{\mathrm{Negative}}\right)}{\left(OD_{\mathrm{Positive}}-OD_{\mathrm{Negative}}\right)}\times 100\%$$

### 2.11. Cell cytotoxicity evaluation

HUVEC cells were seeded into 96-well plates (5 × 10^3^/well) in DMEM at 37 °C in a 5% CO_2_ atmosphere for 24 h. After that, the cells were treated with different concentrations of empty formulations and were incubated for another 24 h. At the end of the incubation, 5 mg/mL MTT PBS solution was added, and the plates were incubated for another 4 h. Then, the MTT-containing medium was replaced with 150 µL of dimethyl sulfoxide (DMSO) per well. Finally, the absorbance of per well was measured using a microplate reader at 490 nm.

### 2.12. In vivo safety evaluation

The healthy mice were randomly divided into four groups (*n* = 3) and treated with saline(control), free CEP, CEP/NLCs, and MM-CEP/NLCs via the tail vein every other day, respectively. The body weights were recorded after each treatment. On the 14th day, all mice were sacrificed and samples of the heart, liver, spleen, lung, and kidney were collected and fixed in paraformaldehyde for hematoxylin and eosin (H&E) staining.

### 2.13. Antiphagocytosis ability of the nanosystems

The *in vitro* antiphagocytosis ability was evaluated in RAW264.7 cells. In brief, cells were seeded into a culture plate at 1 × 10^5^ cells/well and cultured 24 h. Then the cells were incubated with Cou6 labeled NLCs or MM-NLCs. After 2 h, cells were collected for quantification by fluorescence-activated cell sorting (FACS) analysis (FACSAria III, BD, USA). Cells were also stained with DAPI for visualization by confocal laser scanning microscopy (CLSM) (LSM 880, ZEISS, Germany).

### 2.14. Pharmacokinetic studies

To characterize the pharmacokinetics of MM-NLCs *in vivo*, MM-NLCs were prepared from DiR-labeled NLCs. To study pharmacokinetics, 200 μL of 3 mg/mL fluorescently labeled nanoparticles were administered intravenously into male KM mice weighing 20–25 g. Blood samples were collected from the tail at 5 min, 30 min, 1 h, 3 h, 7 h, 24 h, 36 h, 48 h, and 60 h. Samples were then diluted 10 × with PBS and the fluorescence intensity was determined with a microplate reader.

### 2.15. In vivo distribution of MM-NLCs in acute lung injury mouse model

The lipopolysaccharide (LPS) was isolated from the outer membrane of Gram-negative bacteria to induce acute lung injury models (Wang et al., 2014, 2020). LPS (5 mg/kg) solution was dropped into mice via intratracheal instillation. After 5 h, the mice were randomly divided into 3 groups and treated with free DiR and different DiR-labeled formulations via tail vein injection. At 24 h, the main organs were isolated for imaging using an IVIS *in vivo* system (IVIS^®^ Spectrum, PerkinElmer, USA) at 750/800 nm. Living Image^®^ software (Caliper, Alameda, CA) was used to quantify the fluorescence signals.

The specific targeting of MM-NLCs to lung inflammation sites was evaluated under CLSM. DiI-labeled NLCs and MM-NLCs were injected into mice via the tail vein. At 12 h, lung tissues from each group were removed, frozen in OCT, and sectioned at 10 μm (RM2016, Leica, Germany). Meanwhile, samples were stained with DAPI (blue, Beyotime) for visualization. The red signals of nanotherapeutics and blue signals of nuclei were recorded to assess the targeting capacity of MM-NLCs to lung inflammation sites in the acute lung injury mouse model.

### 2.16. In vivo therapeutic efficacy

The acute inflammation lung mouse model was generated as mentioned above. Then, mice were treated with PBS (control), free CEP, CEP/NLCs, and MM-CEP/NLCs via the tail vein (normal group represented normal mice without being challenged with LPS), respectively. Mice were sacrificed 12 h after administration, and bronchoalveolar lavage fluid (BALF) was collected by reference (Kao et al., 2015). The right main bronchus was tied and the left lung was lavaged five times with 0.6 mL aliquots of sterile saline and the BALF was collected. Then centrifuge the BALF at 1500 rpm for 10 min. Resuspend the pellet, count the total number of cells. The levels of TNF-α and IL-6 inflammatory factors in the BALF supernatant were determined by ELISA kits. Meanwhile, protein concentration in the supernation of BALF was determined by the BCA method using a commercial kit according to the protocol. The level of lung water content was analyzed by measuring the ratio of the wet lung to dry lung (wet-to-dry) weights.

### 2.17. Histological examination

After 12 h or 24 h of administration, the mice were sacrificed. Lungs were isolated and fixed with 4% paraformaldehyde, embedded in paraffin, and then sliced and stained with H&E. The samples were observed and photographed under an optical microscope.

### 2.18. Statistical analysis

Experimental data are presented as the means ± standard deviation (SD). The data were analyzed using one-way analysis of variance (ANOVA). The relative fluorescence intensity of at least three samples per group with three different sections per sample were assessed using ImageJ software (NIH Image J system, Bethesda, MD). *p* < .05 indicated a statistically significant difference.

## 3. Result and discussion

### 3.1. Preparation and characterization of nanocarriers

**Figure 1.**
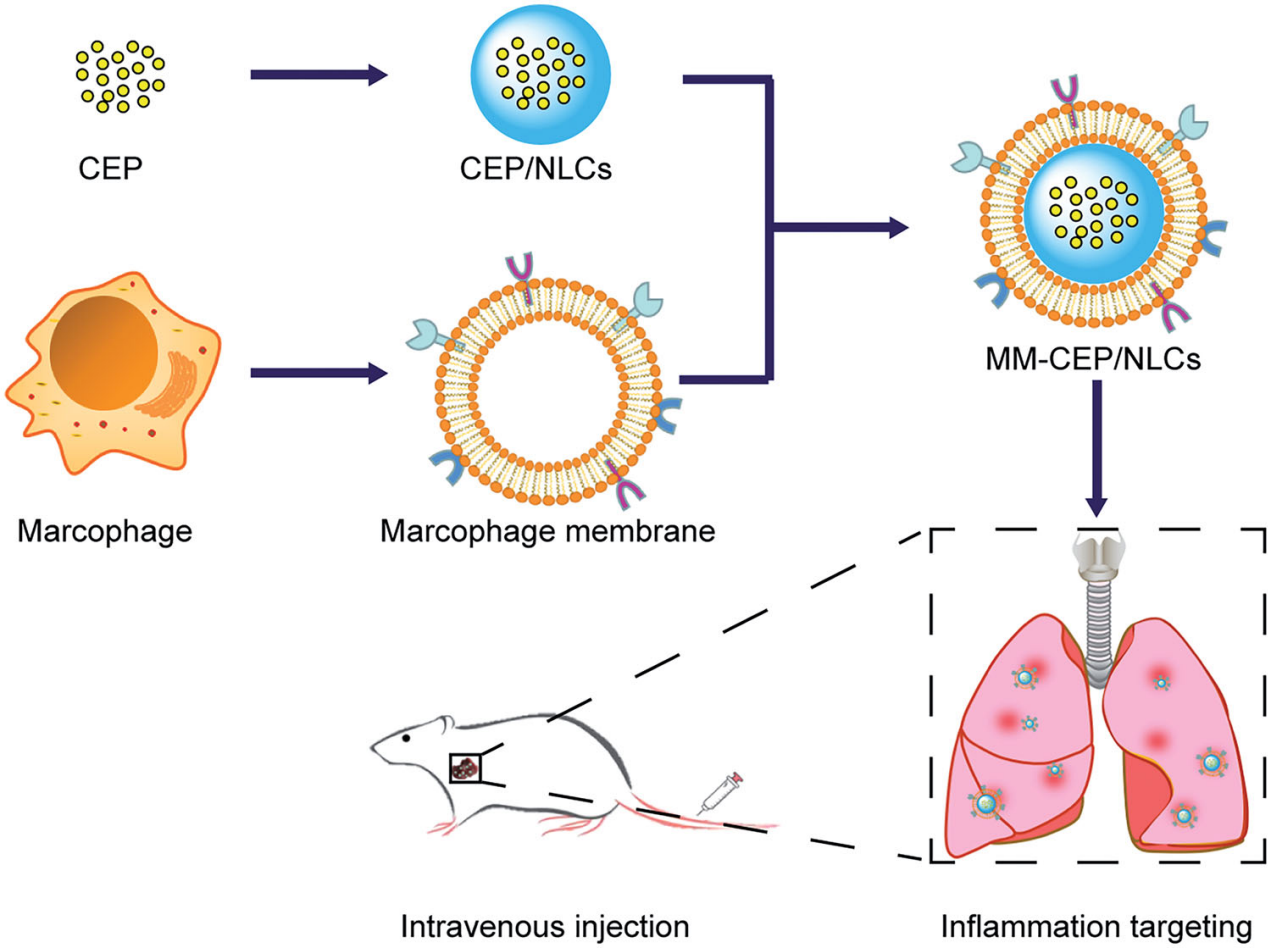
Schematic synthesis of MM-CEP/NLCs and their inflammatory targeting procedure to the ALI mouse. CEP/NLCs are prepared by solvent evaporation-ultrasonic dispersion method. MMs were coated on these CEP/NLCs to fabricate MM-CEP/NLCs. After injected into ALI mouse, these MM-CEP/NLCs can target to the inflammatory sites just like the accumulation of macrophage in lungs when pneumonia burst.

The preparation scheme for MM-CEP/NLCs is illustrated in Figure 1. First, the macrophage membranes (MMs) (‘outer shell’ of the biomimetic nanosystem) were derived from RAW264.7 cells by probe ultrasound method, and nanostructured lipid carriers (NLCs) loading with CEP (‘inner core’ of the biomimetic nanosystem) were prepared using the solvent injection method. Next, the resulting MMs were coated onto the surface of CEP/NLCs through mechanical extrusion to form macrophage membranes-coated nanostructured lipid carriers (MM-CEP/NLCs) with a characteristic core-shell structure. CLSM was used to observe the separation of the nucleus and membrane. As shown in Supplementary Figure S1, after the first centrifugation, the nuclei had been centrifuged out and some cell membranes had also been collected. After the second centrifugation, it was obvious that the nuclei had been removed and the cell membranes were purified.

**Figure 2.**
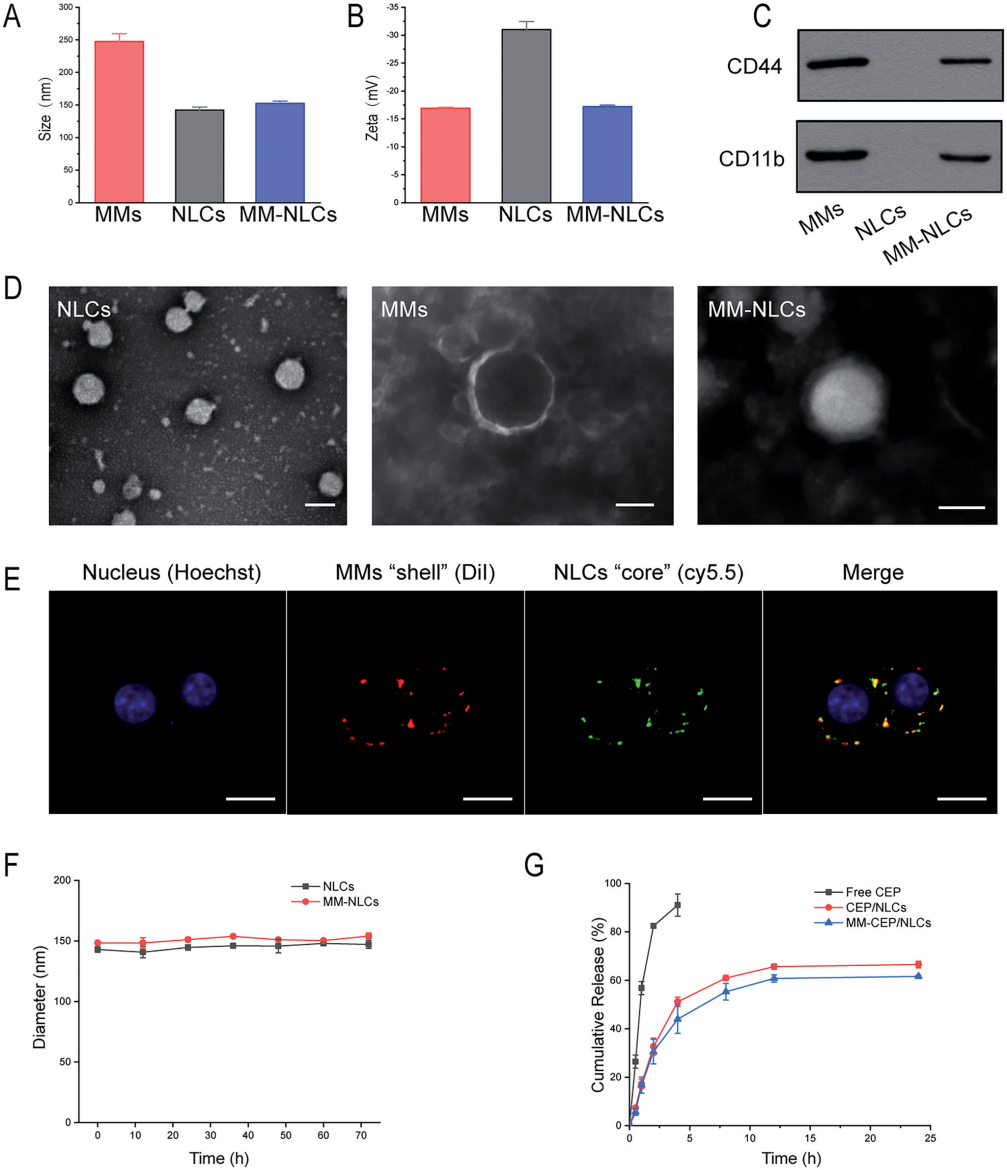
Characterization of the MM-coated biomimetic nanoparticles. (A) The size and (B) zeta potentials of MMs, NLCs and MM-NLCs (*n* = 3, mean ± SD). (C) Western blot results of CD44 and CD11b in MMs, NLCs and MM-NLCs. (D) TEM images of NLCs, MMs, and MM-NLCs (scale bar = 100 nm). (E) CLSM images of the colocalization of the nucleus (blue), MMs ‘shell’ (green) and NLCs ‘core’ (red) (scale bar = 10 μm). (F) Size stability of NLCs and MM-NLCs in solution with 10% FBS. (G) In vitro CEP release from CEP/NLCs or MM-CEP/NLCs in PBS (pH 7.4) at 37 °C.

The size and morphology of NLCs and MM-NLCs were measured by DLS and TEM as shown in Figure 2. It was clearly seen that the average size of NLCs was 142.44 ± 4.3 nm. After the coating of MMs, the average size increased to 152.48 ± 3.69 nm, about 10 nm bigger than that of NLCs (Figure 2(A)). TEM measurements showed that the morphology of NLCs particles was spherical with an average diameter of approximately 150 nm (Figure 2(D)), which was consistent with DLS results. Macrophage membranes could also be obviously observed on the surface of MM-NLCs while only bare nanoparticles were seen in the TEM image of NLCs (Figure 2(D)). The zeta potential of NLCs was −31.0 ± 1.45 mV, while the zeta potential of MMs was −16.93 ± 0.17 mV. After coating MMs on the surface of NLCs, the zeta potential was similar to macrophage membranes (Figure 2(B)), indicating the coverage of MMs on the surface of NLCs.

The successfully coating MMs on the surface of NLCs was further confirmed by the confocal microscopy (Figure 2(E) and Supplementary Figure 3). Cy5.5 (green) encapsulated NLCs were coated with DiI (red) labeled MMs and were observed in captured images. Yellow color was observed when these two images were overlapped, illustrating that the green color and red color were in the same particle, proving that NLCs were successfully covered by MMs. In addition, the protein profiles in the MMs and MM-NLCs were determined by SDS-PAGE. The protein composition in the MMs was mostly retained in the MM-NLCs, but no protein signal was detected from the NLCs (Supplementary Figure S2), suggesting the successful translocation and retention of natural macrophage cell membranes onto the NLCs surface. Specific membrane proteins are signs of the functional integrity of the cell membrane. According to previous reports, CD44 and CD11b are related to the adhesion and signal transduction in inflammation (Worthen et al., 1989; Li et al., 2019). Furthermore, the inflamed endothelium highly expresses P-selectin and intercellular cell adhesion molecule-1 (ICAM-1), which are corresponding ligands to CD44 and CD11b (Bullard et al.,^1999^; Volin, 2005). Western blot analysis was carried out to confirm that the expression of CD44 and CD11b was maintained in the MM-NLCs, similar to that of MMs (Figure 2(C)). Collectively, all these representative proteins could be found in MMs and MM-NLCs, which indicated that the coating process did not affect the biological properties of MMs.

Because stability was a prerequisite for further applications *in vivo*, the agglomerations of the nanosystem in PBS with 10% FBS were evaluated within 72 h at 37 °C to mimic *in vivo* conditions. The results indicated that both NLCs and MM-NLCs exhibited good stability in a solution containing 10% FBS (Figure 2(F)). The release kinetics of CEP from CEP/NLCs and MM-CEP/NLCs were investigated in buffer solutions that simulated the extracellular environment (PBS, pH 7.4). As shown in Figure 2(G), both CEP/NLCs and MM-CEP/NLCs demonstrated sustained release behaviors at normal physiological pH. After 24 h of incubation, 66.53% and 61.66% of CEP was released from CEP/NLCs and MM-CEP/NLCs, respectively. Compared to CEP/NLCs, MM-CEP/NLCs showed a slightly slower CEP release profile. In general, the steady and long-term CEP release behavior of MM-CEP/NLCs indicated their potential to be used for sustained drug release.

### 3.2. Biosafety assessment

**Figure 3.**
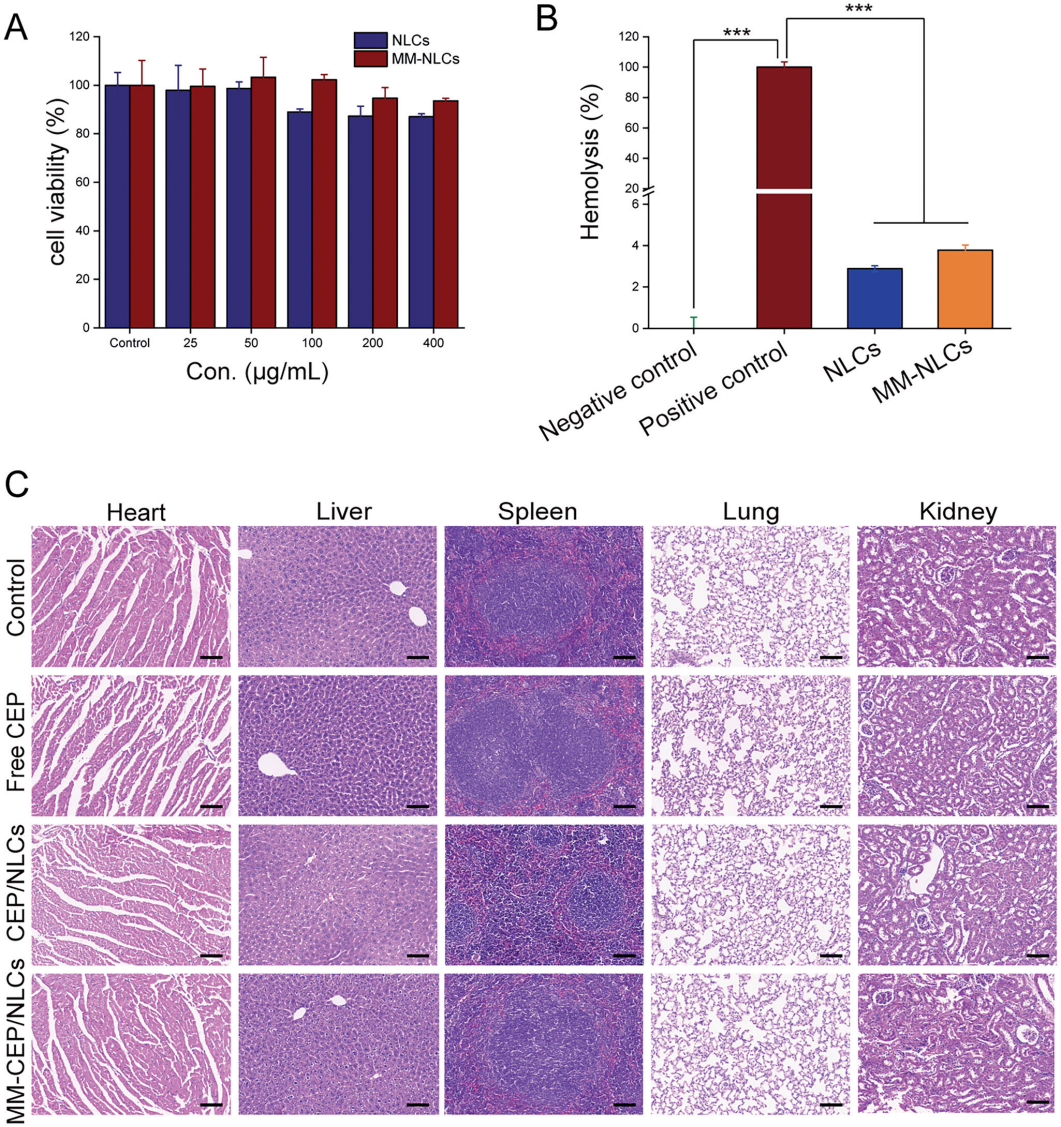
Preliminary safety evaluation of the biomimetic nanosystem. (A) Cell viability of HUVEC cells incubated for 24 h with different concentration of empty NLCs and MM-NLCs (*n* = 3, mean ± SD). (B) Hemolysis assay of empty formulations. 0.9% (w/v) sodium chloride solution and double distilled water were served as the negative and positive control, respectively (*n* = 3, mean ± SD, ***p < .001). (C) Histological staining of organs from healthy mice treated with different CEP-load formulations (scale bar = 100 μm).

In addition to having suitable physiochemical properties, an ideal nanocarrier should have minimal toxicity and good blood compatibility. Therefore, the cytotoxicity of empty NLCs and MM-NLCs in HUVEC cells was investigated. As shown in Figure 3(A), the toxicity results indicated that elevated levels of empty NLCs caused slight side effects on HUVEC cells. While, even in elevated levels, the viabilities in the HUVEC cells were higher than 90% after incubation with the detected doses of the empty MM-NLCs for 24 h, revealing that MM-NLCs were relatively safe. Additionally, the hemolysis of MM-NLCs was detected by the direct contact method (Bender et al., 2012). The visual hemolytic images showed no significant hemolysis of either NLCs or MM-NLCs (Supplementary Figure S4). As shown in Figure 3(B), the percentage of hemolysis increased to about 2.5–4.0% after treatment with empty novel NLCs and MM-NLCs compared with that of the control, indicating that these formulations had good blood compatibility (less than 5%). MM-NLCs had higher hemolysis than NLCs, possibly because the macrophage membrane was isolated from murine monocyte/macrophage cell line, while the red blood cells used in the hemolysis experiment are from rats, and there are species differences.

Furthermore, KM mice (*n* = 3) received an i.v. injection of saline, free CEP, CEP/NLCs, or MM-CEP/NLCs to evaluate *in vivo* toxicity. On the 14th day after the injection, all mice were euthanized and their major organs were collected for histology analysis (Figure 3(C)). Compared with the saline (control) group, no indicators of damage were observed for these organs after treatment, suggesting that CEP solution, CEP/NLCs, and MM-CEP/NLCs did not induce tissue toxicity at the current CEP dosage. All of the above results suggested a good *in vivo* biocompatibility of MM-CEP/NLCs.

### 3.3. Long circulation feature

**Figure 4.**
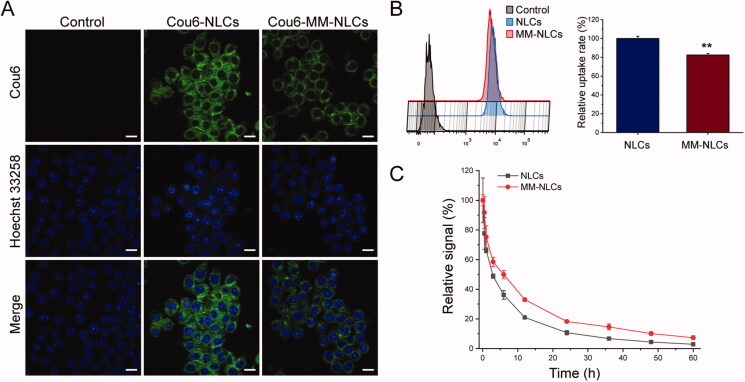
Long circulation feature. (A) CLSM images of RAW 264.7 cells after incubation with Cou6-tagged NLCs and MM-NLCs (scale bar = 10 μm). (B) FACS results and quantification of cellular uptake of NLCs and MM-NLCs in RAW264.7 cells (*n* = 3, mean ± SD, **p < .01). (C) Relative fluorescence intensity of NLCs and MM-NLCs in blood (*n* = 3, mean ± SD).

RES evasion ability of nanosystem is crucial to extend the residential time during systemic circulation to achieve targeting delivery. To verify whether MM-NLCs inherit the immunological characteristics of macrophages, the *in vitro* antiphagocytosis assay was first conducted. RAW264.7 cells were utilized as a cell model to reckon the stealth power of the biomimetic nanosystem (Xuan et al., 2016; Yao et al., 2021). The captured images showed that a less fluorescence level in macrophages treated with MM-NLCs than in the bare NLCs group (Figure 4(A)). The FACS analysis showed that the internalization of MM-NLCs in macrophages was significantly reduced compared with the NLCs group (Figure 4(B)). Accordingly, the MMs coating may effectively block nonspecific phagocytose by macrophages. Accumulating evidence has suggested that the ability of macrophage membrane-coated nanoparticles to survive in macrophages is due to a collective contribution of the proteins and glycosyl groups on the cell membrane surface. Specifically, CD47, a protein embedded in the macrophage membrane, can inhibit macrophage phagocytosis by bonding with the SIRP-α receptor (Wang et al., 2021).

After a series of *in vitro* studies, a pharmacokinetics study was employed to investigate the *in vivo* behavior of formulations. It was found that MM-NLCs showed initially high blood circulating levels, while bare NLCs were more quickly cleared from the systemic circulation (Figure 4(C)). At 24 h, NLCs and MM-NLCs showed 10.7% and 18.2% retention in the blood, respectively. Therefore, the MM-NLCs exhibited superior blood retention, suggesting that MMs inheriting the characteristics of natural macrophages could efficiently prolong the blood circulation time to potentially enhance targeted drug delivery.

### 3.4. *In* vivo *targeting*

**Figure 5.**
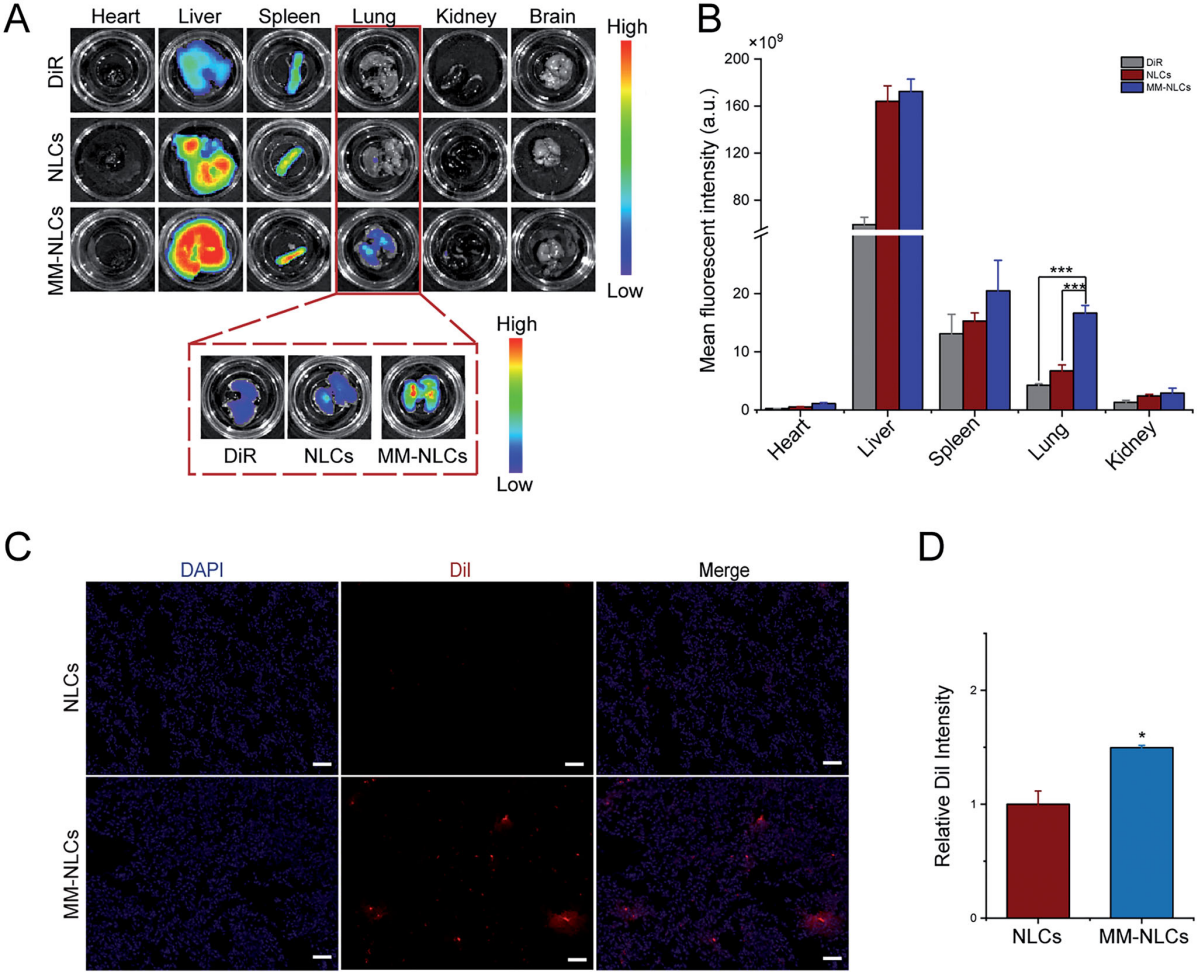
*In vivo* targeting evaluation. (A) Representative *ex vivo* fluorescence images and (B) quantitative data of DiR fluorescent signals accumulated in lung at 24 h (*n* = 3, mean ± SD, ***p < .001). (C) CLSM images of accumulated MM-NLCs in lung at 12 h (scale bar = 50 μm). (D) ImageJ was utilized to quantify the distribution of NLCs and MM-NLCs in lung (*n* = 3, mean ± SD, *p < .05).

The effect of macrophage membrane decoration on *in vivo* biodistribution was evaluated in the acute lung injury models induced by LPS. NLCs and MM-NLCs were marked with DiR for *ex vivo* imaging. After 12 h or 24 h of intravenous administration, the mice were sacrificed, and their main organs were harvested and processed *ex vivo*. The captured images showed that NLCs, as well as MM-NLCs, were mainly distributed to the liver, spleen, and lung at 12 h or 24 h (Figure 5(A) and Supplementary Figure S5(A)). The MM-NLCs accumulated in the lung sites could be clearly observed by *ex vivo* imaging (Figure 5(A)). The fluorescence quantitative results showed that the fluorescence intensity of the liver, spleen, and kidney in the MM-NLCs group was not significantly different from that in the NLCs group. Besides, MM-NLCs treated mice had much higher fluorescence intensity in lungs among all these organs. At 12 h and 24 h, the intensity of the fluorescence signals in the lung from MM-NLCs treated group was enhanced 1.54-fold and 2.47-fold in comparison to that of the NLCs group, respectively (Figure 5(B) and Supplementary Figure S5(B)). In terms of NLCs from 12 h to 24 h, the fluorescence intensity of all organs decreased except liver, while the fluorescence intensity of MM-NLCs still remained at a high level. These results revealed that MM-NLCs had a longer lifetime and superior lung targeting ability *in vivo*. Furthermore, the inflammatory mouse lung targeting of MM-NLCs also was visualized under CLSM. As shown in Figure 5(C), the red fluorescence signals of MM-NLCs were readily detected with strong intensity in lung tissue. However, in the NLCs-treated group, only a few red spots could be observed in lung tissue, suggesting MM-NLCs had better lung targeting ability. ImageJ quantitation also indicated MM-NLCs had better distribution in lung tissues (Figure 5(D)). It was mainly because MM-NLCs which inherited functional proteins of macrophage membranes can actively target dysfunctional endothelium (Tang et al., 2019). Moreover, the EPR effect also exists in acute lung injured lesions based on the leaky endothelium from inflammation (Wang et al., 2021). Overall, MM-CEP/NLCs with long circulation and specific interactions with dysfunctional endothelium have the ability to efficiently target and accumulate in lung inflammatory sites.

### 3.5. In vivo therapeutic efficacy

**Figure 6.**
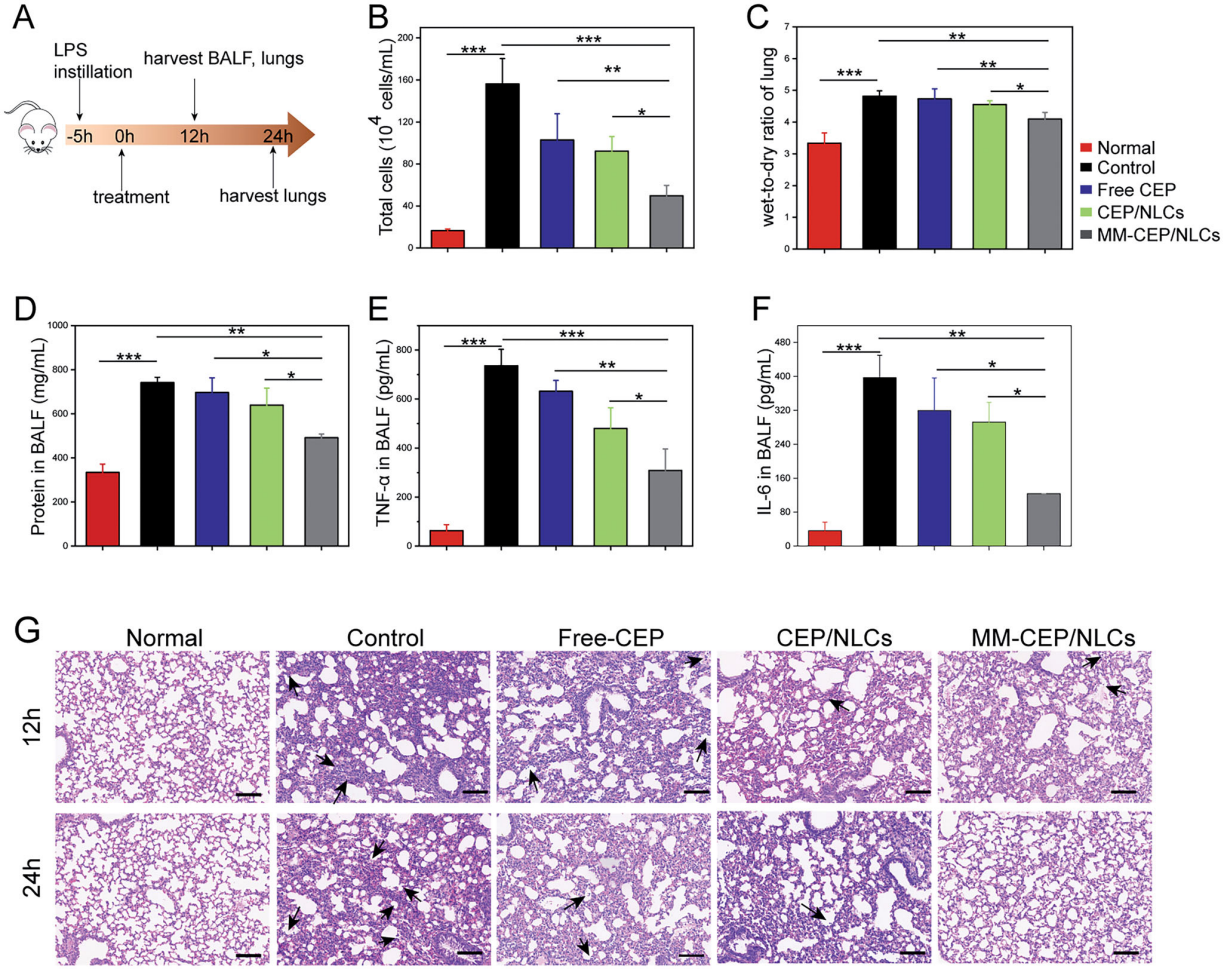
MMs-coated biomimetic nanoparticles can improve the therapeutic efficacy of ALI in a mouse model. (A) Experimental protocol for evaluating the therapeutic effect of free CEP, CEP/NLCs and MM-CEP/NLCs. (B) Total cell counts in BALF. (C) Lung water content was assessed by the measurement of wet-to-dry ratio. (D) Protein contents in BALF were measured using a BCA kit. (E) TNF-α and (F) IL-6 in BALF were measured by ELISA kit. (G) Hematoxylin and eosin (H&E)-stained lung tissue sections were imaged (scale bar = 100 μm). (*n* = 4, mean ± SD, *p < .05, **p < .01, ***p < .001 and ns, no significance).

MM-CEP/NLCs were further evaluated for their protective effects in a mouse model of ALI induced by intratracheal injections of LPS. Five hours after the LPS challenge in the mouse lung, PBS (control), free CEP (5 mg/kg), CEP/NLCs, and MM-CEP/NLCs were injected via tail vein (Figure 6(A)). In animal models, intratracheal administration of LPS causes lung inflammatory cytokines production, inflammatory cell recruitment, increased protein permeability, and extensive morphological damages including edema, hemorrhage, the thickness of alveolar walls, and infiltration of neutrophils in alveolar and interstitial spaces (Zhang et al., 2019).

First, compared to the normal group, LPS injured mice with the treatment of PBS had highly elevated lung water content and cell number, protein concentration, and the level of inflammatory cytokines TNF-α and IL-6 in the BALF (Figure 6(B–F)). For the mice treated with free CEP, CEP/NLCs or MM-CEP/NLCs, the severity of these indexes was attenuated in different degrees. Notably, compared with free CEP or CEP/NLCs treatment, the lung water content, cell number, protein concentration, inflammatory factors TNF-α and IL-6 in BALF were significantly reduced after treatment with MM-CEP/NLCs, indicating that inflammation was effectively reduced. These results indicated that the lung inflammation was resolved and lung vasculature was repaired.

Next, the therapeutic efficacy of various drug formulations was evaluated at a tissue level by examining the pathological changes of the lung during the treatment (Figure 6(G)). Compared to the healthy mice, the control group exhibited severe acute lung injury. In terms of the control group from 12 h to 24 h, the inflammation became severer as more and more alveoli were ruptured and filled with inflammatory cells (black arrows). For free CEP and CEP/NLCs treated groups, the degree of inflammation was relieved slightly as intact alveoli could be seen in the sections and the amount of inflammatory cells was reduced along with the time extension. However, after treatment with MM-CEP/NLCs, leukocyte infiltration was significantly decreased compared with the other groups in the lung section from mice from 12 h to 24 h. At the time point of 24 h, there were almost full of intact alveoli in the image of the pathological section instead of inflammatory cells, which meant the remarkable efficacy of MM-CEP/NLCs in targeting ALI treatment.

Dysregulated inflammation and increased lung permeability are the features of ALI (Mehta & Malik, 2006; Matthay et al., 2012). The macrophage membrane coating strategy endows MM-CEP/NLCs with the functions of long-term circulation (Figure 4) and active targeting to the dysfunctional endothelium (Figure 5), allowing MM-CEP/NLCs to efficiently accumulate at lung inflammation sites. Then, the loaded CEP is released from the MM-CEP/NLCs, thereby increasing the local drug concentration to inhibit the inflammatory responses in the lesion, finally significantly attenuating the progression of ALI. In addition, recent studies have demonstrated that macrophage membrane coating allows nanoparticles to neutralize LPS and suppress the secretion of inflammatory factors by inhibiting the LPS mediated signaling pathway (Thamphiwatana et al., 2017; Ou et al., 2020). It might be one of the reasons why MM-CEP/NLCs have a significant therapeutic effect on ALI.

Collectively, the above results suggested that MM-CEP/NLCs owned superior advantages in the treatment of lung inflammation compared with other groups. These *in vitro* and *in vivo* results suggested that the superiority of MM-NLCs investigated for the physiochemical properties (Figure 2), safety (Figure 3 and Supplementary Figure S4), long circulation feature (Figure 4), inflammatory sites targeting (Figure 5 and Supplementary Figure S5). The CEP-loaded biomimetic engineered delivery nanosystem is a potential therapeutic candidate to treat lung inflammation in ALI therapy.

## 4. Conclusions

In summary, our research here presented a kind of macrophage membranes-coated nanoparticles that can mimic the property of macrophage to accumulate at the inflammatory tissue. The biological features of outer macrophage membranes provided favorable biocompatibility and RES evasion in system circulation for the nanosystem. Additionally, the superiority of MM-CEP/NLCs was designed at the animal level and showed a good therapeutic effect on ALI. Therefore, this biomimetic strategy could have great potential in the design of inflammatory targeting delivery systems and may be applicable to treat different kinds of inflammation.

## Supplementary Material

Supplemental Material
