# Supplementary material for: Cepharanthine loaded nanoparticles coated with macrophage membranes for lung inflammation therapy
Source: Drug Deliv. 2021 Dec 6;28(1):2582–93. doi: 10.1080/10717544.2021.2009936 (PMC8654408; doi:10.1080/10717544.2021.2009936)
Supplement: Supplemental Material [file IDRD_A_2009936_SM5971.zip › Supplementary_materials_caption.docx]

**Supplementary materials**

**Fig. S1**. Isolate cell membranes after fluorescent staining. (A) RAW264.7 cells. (B) Cell nuclei obtained by centrifugation in the cell membrane preparation. (C) Cell membranes after purification. Hoechst 33258 (blue), DiI (red), (scale bar = 20 μm).

**Fig. S2**. SDS-PAGE protein analysis of MMs, NLCs and MM-NLCs.

**Fig. S3**. Fluorescence images of the colocalization in HUVEC cells at 37°C for 2h: Hoechst 33258 stained nuclei (blue); DiI labeled macrophage membrane (red); the nucleus (blue); Cy5.5 labeled NLCs core (scale bar = 10 μm).

**Fig. S4**. Images of the hemolysis test with NLCs and MM-NLCs.

**Fig. S5**. (A) Representative *ex vivo* fluorescence images and (B) quantitative data of DiR fluorescent signals accumulated in lung at 12 h (n = 3, mean ± SD).

**Fig. S6**. (A) Representative H&E-stained pathological sections of lungs at (A) 12 h, (B) 24 h after the LPS infected mice treated with PBS, free CEP, CEP/NLCs and MM-CEP/NLCs, respectively (scale bar = 100 μm).
