# Supplementary figures and images for: Cepharanthine loaded nanoparticles coated with macrophage membranes for lung inflammation therapy
Source: Drug Deliv. 2021 Dec 6;28(1):2582–93. doi: 10.1080/10717544.2021.2009936 (PMC8654408; doi:10.1080/10717544.2021.2009936)

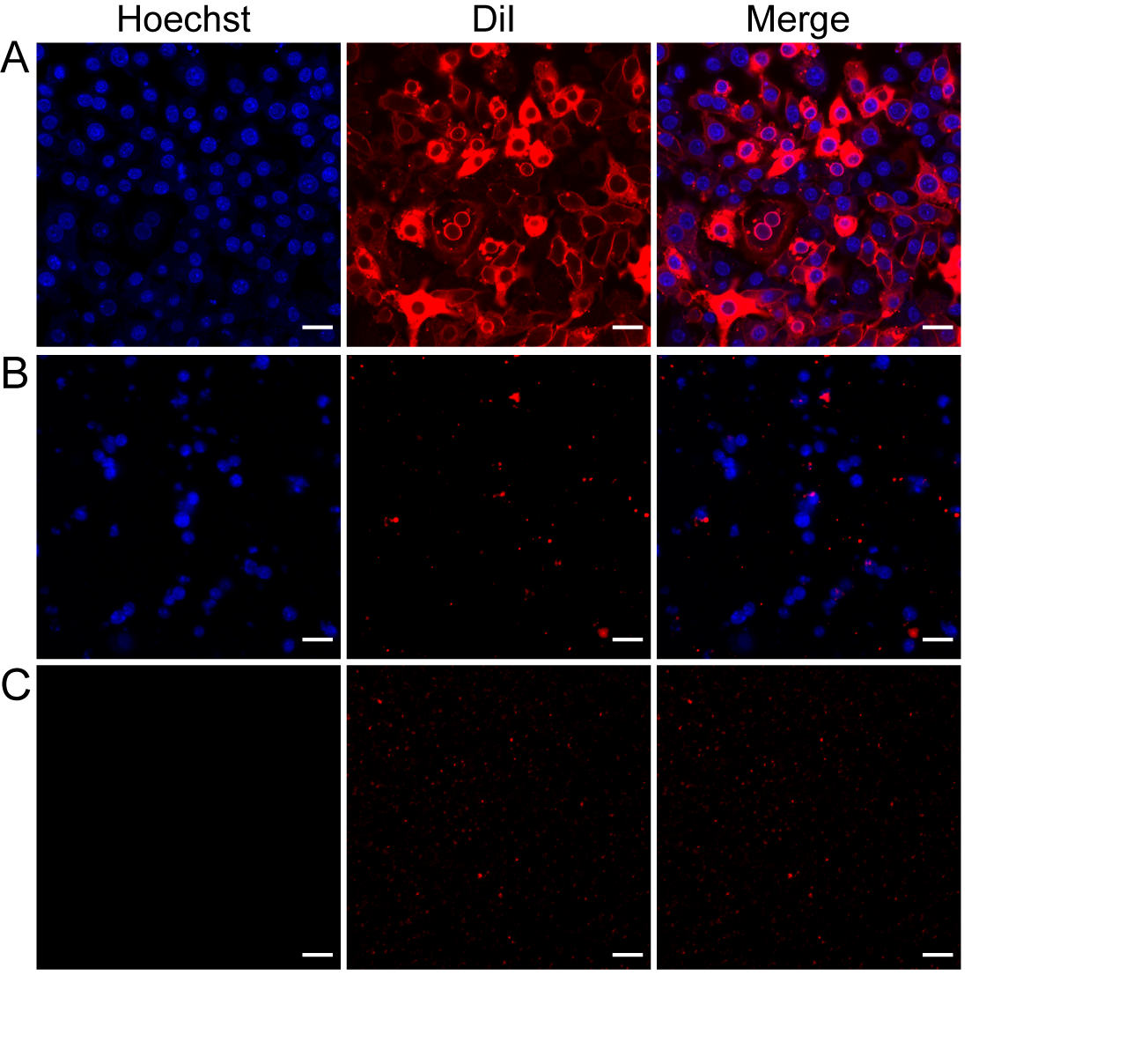

Supplement: Supplemental Material [file IDRD_A_2009936_SM5971.zip › Figure_S1.tif]

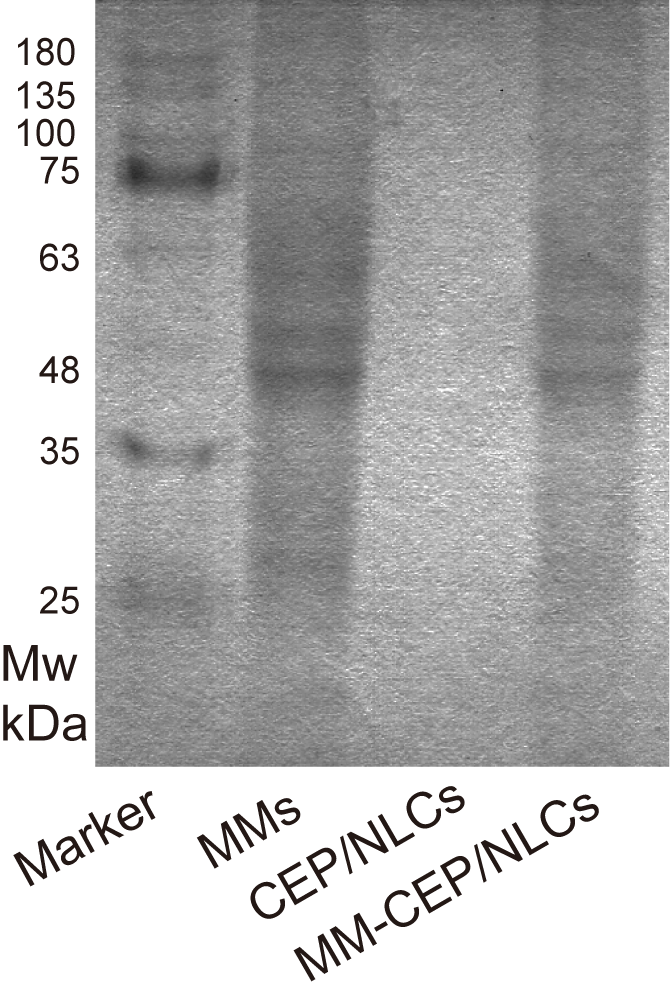

Supplement: Supplemental Material [file IDRD_A_2009936_SM5971.zip › Figure_S2.tif]

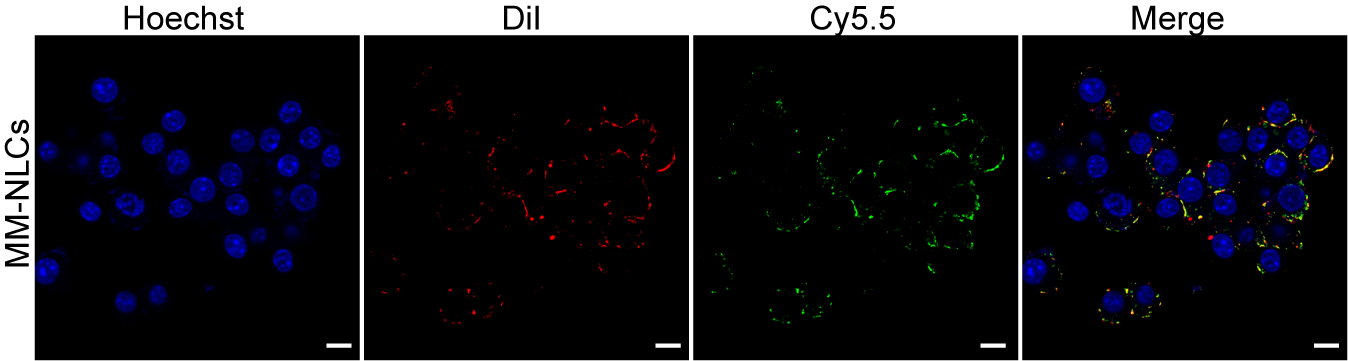

Supplement: Supplemental Material [file IDRD_A_2009936_SM5971.zip › Figure_S3.tif]

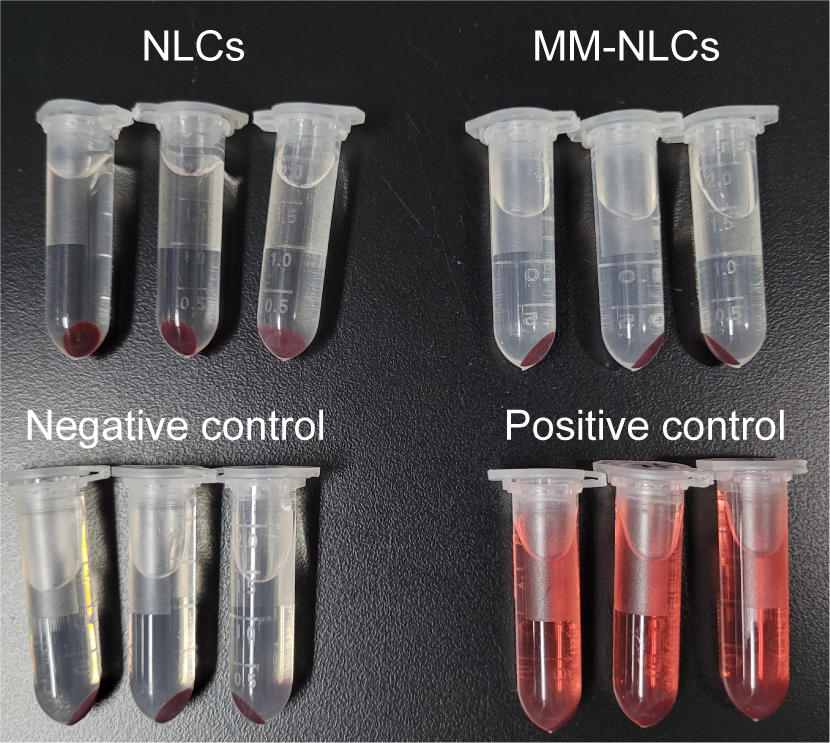

Supplement: Supplemental Material [file IDRD_A_2009936_SM5971.zip › Figure_S4.tif]

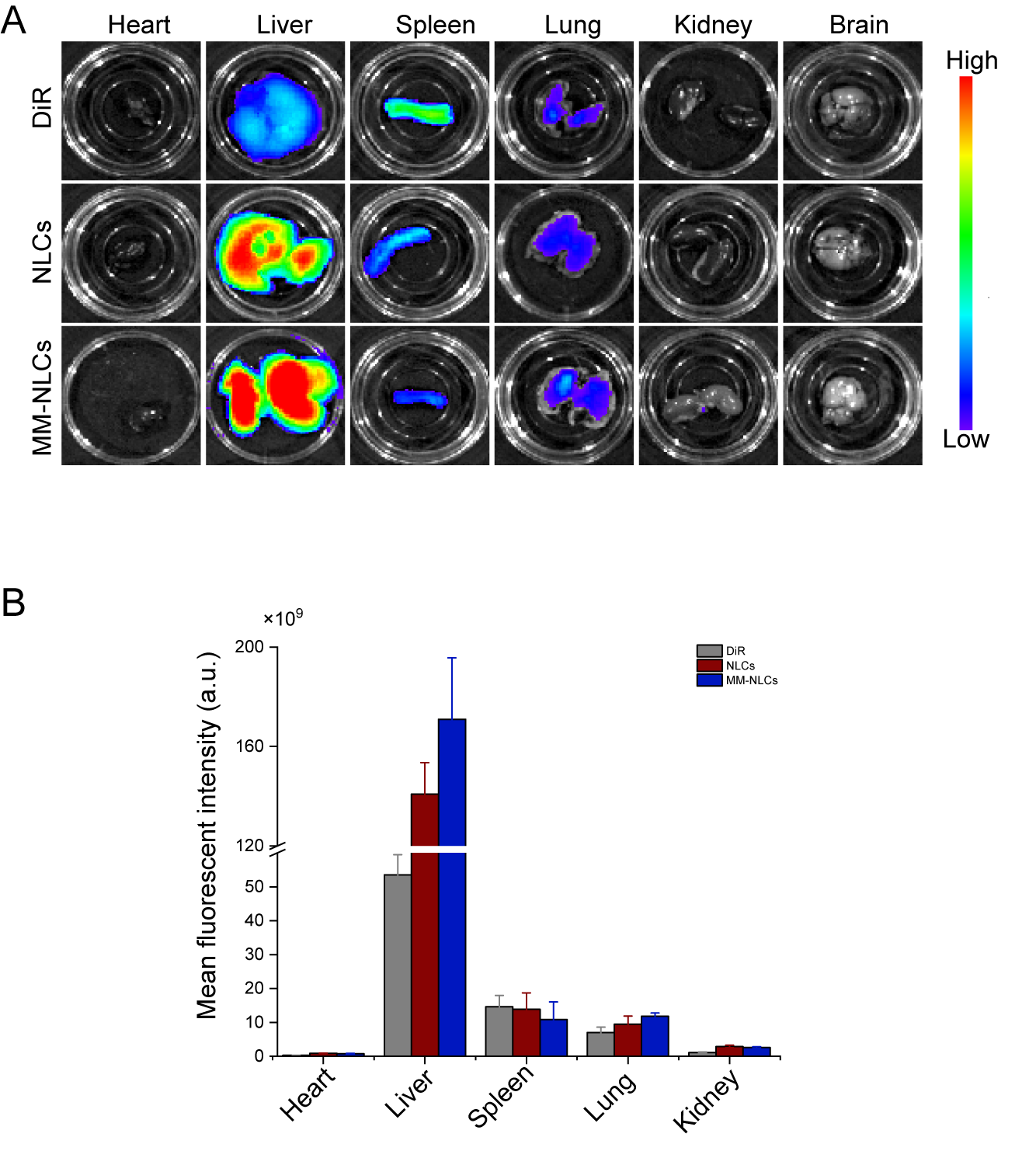

Supplement: Supplemental Material [file IDRD_A_2009936_SM5971.zip › Figure_S5_revision.tif]

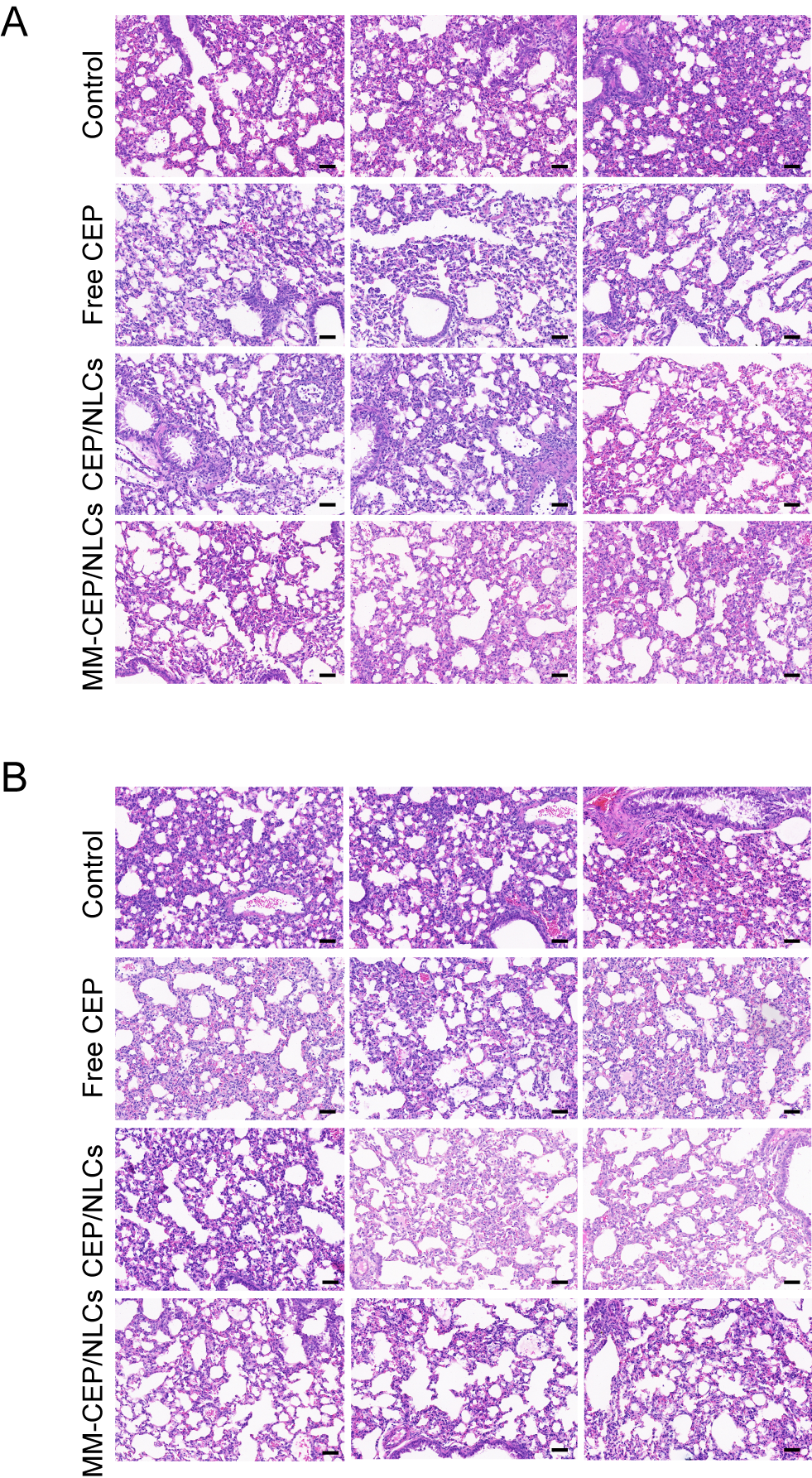

Supplement: Supplemental Material [file IDRD_A_2009936_SM5971.zip › Figure_S6.tif]
